# Validation of a triiodothyronine (T3) ELISA for mouse fecal samples

**DOI:** 10.14814/phy2.70115

**Published:** 2024-10-29

**Authors:** Lucia M. Thompson, Brailey M. Coulter, Cinnamon L. VanPutte

**Affiliations:** ^1^ Biological Sciences Southern Illinois University Edwardsville Edwardsville Illinois USA; ^2^ Biomedical and Craniofacial Sciences Southern Illinois University School of Dental Medicine Alton Illinois USA

**Keywords:** fecal extracts, mice, non‐invasive sampling, periodontal disease, thyroid hormones

## Abstract

Acquiring sufficient blood for hormone analysis in mice can be a limiting step. Hormone analysis techniques using non‐invasive sample collection have been vigorously developed for endangered species, from whom blood sampling is prohibited, or from species that are otherwise difficult to handle in a laboratory setting. Because there are interactions between glucocorticoids and thyroid hormones (T3 and T4), reducing the animal's “distress” during sample collection is imperative. Measurement of fecal T3 provides less sensitive, baseline information regarding thyroid function while permitting a non‐invasive technique for more frequent sampling. We demonstrated that using a methanol extraction protocol produced the most reliable fecal T3 measurement in an enzyme‐linked immunosorbent assay (ELISA). We found that during a thyroid hormone‐treated state, fecal and plasma T3 measurements from mice are directly related, while during a methimazole‐treated state, fecal and plasma T3 measurements from mice are inversely related. Fecal samples are a useful way to monitor thyroid hormone function in laboratory mice.

## 1 INTRODUCTION

*Mus musculus* have been utilized as a mammalian research model for over 100 years. The accumulated knowledge base of mouse genetics, physiology, and disease is vast. In fact, scientists know more about mouse health and disease than most other animal research models, except for humans. At multiple levels, mouse biology is strikingly similar to human biology. Additionally, mice have many other characteristics that make them a desirable model system, including their small size, making the mouse a fiscally friendly model organism. Mice can be easily bred and are often inbred to reduce genetic variability among individuals. However, despite all the desirable characteristics of mice, they have some challenges when studying physiology. For example, acquiring sufficient blood for hormone analysis is a limiting characteristic. Traditionally, the principal material for hormone analysis has been blood. However, a 25 g mouse has a total blood volume of 1.5 mL, and within the scope of many guidelines, no more than 10% of total blood volume may be collected at one time (Mcguill & Rowan, 1989). Further, this volume may only be acquired every 2–4 weeks. This presents two problems: (1) having sufficient plasma for many commercial enzyme‐linked immunoassays (ELISAs) (50–100 μL) and (2) being able to collect serial samples more frequently than every 2–4 weeks from a single animal. Monitoring concentration changes of several hormones over time using such ELISAs is not feasible.

Our lab is examining the role of thyroid hormone physiology in periodontal disease using C57Bl/6J mice as our model organism. We are specifically interested in monitoring triiodothyronine (T3) and thyroxine (T4) concentrations. Because we are a small, newly minted lab, we are fiscally conservative, which limits the number of animals, and thus the amount of blood, we have for each experiment. This led us to pursue other means for measuring hormone levels. Because both thyroid hormones, T3 and T4, are lipid‐soluble molecules, we began searching the literature for methods such as those used to measure steroid hormones from biological materials other than blood, including feces, urine, and saliva, among others.

Indeed, about three decades ago, so‐called non‐invasive hormone measurement techniques were vigorously developed. In 2010, Wasser et al. published a pivotal paper describing a protocol for measuring fecal thyroid hormones in several mammalian and avian species. In that study, the measurement of fecal T3 was determined to be more informative of the biological activity of thyroid hormones since T4 can be stored for substantial periods of time and is not excreted as rapidly as T3 (Wasser et al., 2010). Several other research groups have optimized similar protocols for monitoring fecal T3 levels in endangered species, from whom blood sampling is prohibited, or species that may be difficult to handle in a laboratory setting. These include Hawaiian monk seals, gray whales, and tigers, among others (Behringer et al., 2023; Cristóbal‐Azkarate et al., 2016; Farmer, 2024; Gesquiere et al., 2018; Gobush et al., 2014; Hu et al., 2018; Hunninck et al., 2020; Jesmer et al., 2017; Keech et al., 2010; LaDue et al., 2023; Lemos et al., 2020; Li et al., 2021; Mondol et al., 2020; Pasciu, Nieddu, et al., 2022; Pasciu, Sotgiu, et al., 2022; Schaebs et al., 2016; Torrico, 2023).

The secretion of thyroid hormones is regulated by a central hypothalamic–pituitary‐thyroid (HPT) axis, which is strongly influenced by circadian rhythms, as seen in amphibians, birds, and mammals (Ikegami & Yoshimura, 2017). It has been reported that fecal thyroid hormone measurements do not reflect the same oscillatory fluctuations as plasma measurements (Behringer et al., 2018). It is also well established that there are interactions between glucocorticoids and thyroid hormones (Benker et al., 1990; Nicolaides et al., 2014; Nicoloff et al., 1970). Given that thyroid hormones' major functions are key to mediating stress‐induced reactions, such as promoting glucose availability, reducing the animal's “distress” during sample collection is imperative. Thus, the measurement of fecal T3 provides less sensitive, baseline information regarding thyroid function while permitting a non‐invasive technique for more frequent sampling.

Thyroid hormones are uniquely suited for extraction from feces. After synthesis and secretion from the thyroid gland, T4 undergoes enterohepatic metabolism, from which bile transports thyroid hormones and metabolites to be excreted through feces (DiStefano III, 1988; Taurog et al., 1952; Van Middlesworth et al., 1971). Specifically, about 20%–30% of secreted T4 and T3 is excreted in rodent feces (DiStefano III & Sapin, 1987). Because thyroid hormones are lipophilic, these hormones can be extracted from fecal samples using organic solvents.

Organic solvent extractions have been validated to measure fecal thyroid hormones in laboratory rabbits, as well as to measure fecal glucocorticoids and sex steroids in laboratory mice (Auer et al., 2020; Chmurska‐Gąsowska et al., 2021; Touma et al., 2004). Radioimmunoassay (RIA) and high‐performance liquid chromatography (HPLC) are commonly used for the quantification of fecal hormones, but these methods can be expensive, more time consuming, and less accessible than a commercially prepared ELISA kit. To our knowledge, no protocol has been validated for the alcohol‐based extraction of T3 from the feces of laboratory mice to be analyzed via ELISA.

To assess whether fecal T3 measurements are a reliable metric of overall thyroid function, it is important that this technique recognizes perturbations to normal thyroid function. Therefore, as biological validation we administered T3 and T4 to laboratory mice to model a hyperthyroid condition. Similarly, we administered methimazole (MMI) to mice to inhibit thyroid hormone production as a model for a hypothyroid condition. The aim of this study was to validate an alcohol‐extraction protocol for use in a commercially prepared ELISA and to assess if fecal T3 measurements from such an assay could reflect overall thyroid function.

## 2 MATERIALS AND METHODS

### 2.1 Animals

Both male and female C57Bl/6J mice were purchased from The Jackson Laboratory (Bar Harbor, ME, USA; RRID:IMSR_JAX:000664). Mice were maintained in a 14 L:10D photoperiod at 25°C, and a maximum of six mice were housed in a single static cage SuperRat 1400 AllerZone™ Micro‐Isolator® unit containing irradiated bedding (SSP ALPHA DRI‐IRRAD). Irradiated 53WU PICOLAB Rodent 20 feed, shipped in contamination prevention packaging, was fed to animals ad libitum. Animal care procedures and experiments designed for this study were approved by the Institutional Animal Care and Use Committee of Southern Illinois University Edwardsville (IACUC protocols #1167/2570).

### 2.2 Sample collection, storage, and extraction

Mice were individually placed in clean cages until defecation occurred. Five fresh fecal pellets were collected using sterile forceps, placed in clean microcentrifuge tubes, and stored on ice. Because fecal T3 measurements will not reflect circadian fluctuations (Behringer et al., 2018), fecal samples can be collected any time of the day. Samples were then stored at −80°C. Lyophilization of fecal samples did not improve T3 yield compared to freshly frozen fecal samples.

Due to the lipid soluble nature of T3, an organic solvent is needed to extract it from fecal samples. For initial trials, 70% ethanol, 80% methanol, and 99.9% methanol were assessed as potential solvents. These are commonly used solvents for similar protocols (Hunninck et al., 2020; LaDue et al., 2023; Pasciu et al., 2024; Wasser et al., 2010) and are widely available. 99.9% methanol was determined to be the most effective solvent for the extraction of T3 from mouse fecal samples.

**FIGURE 1:**
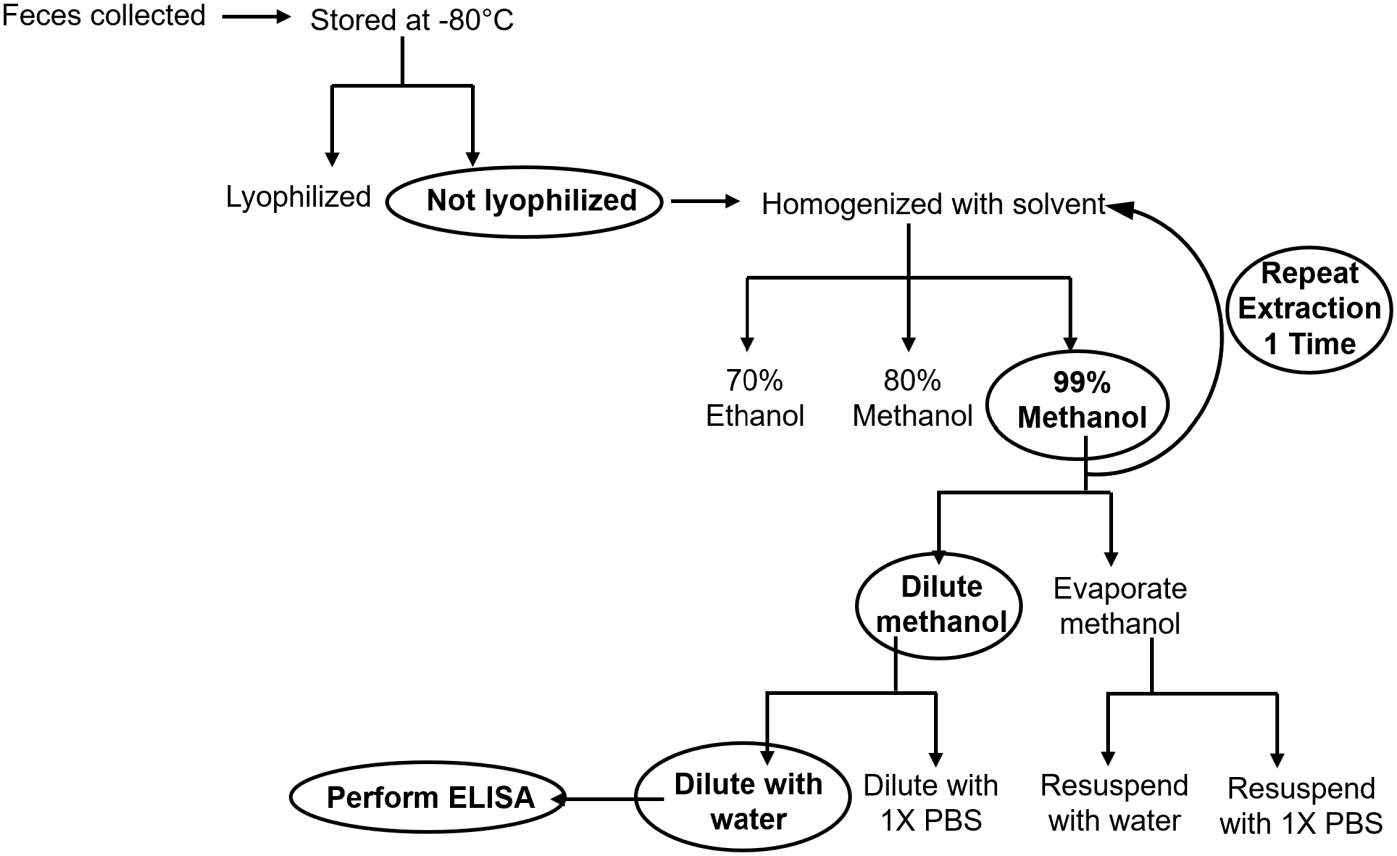
Schematic of experimental design for the optimization of sample storage and extraction for the measurement of fecal T3 from laboratory mice. Circles indicate final protocol steps.

We have found that a ratio of 0.01 g of frozen feces (~1 pellet) to 750 μL of 99.9% methanol (Catalog AC124790025; Fisher Scientific, Pittsburgh, PA, USA) is sufficient for T3 extraction. The mixture of feces and methanol was homogenized using the following steps: (1) manually crushing the fecal pellet with a glass rod, (2) vortexing for 30 s, and (3) sonicating for 30 s at 30 kHz. Samples were kept on ice between steps to avoid hormone degradation. After sonication, samples were centrifuged for 20 min at 2200 rpm at 4°C to remove particulates. After 20 min of centrifugation, the supernatant (supernatant 1) was collected and placed on ice. An additional 750 μL of 99.9% methanol was added to the sample, vortexed, and centrifuged for another 20 min at 2200 rpm. The second supernatant (supernatant 2) was collected, combined with supernatant 1, and stored at 4°C. Evaporation and resuspension of these supernatants greatly reduced T3 yield. Dilution of the methanol‐fecal extract with purified water was determined to produce the most reliable T3 measurement as this prevented a potential matrix effect of excess methanol in an ELISA. A flow chart of the final protocol is shown in Figure 1.

### 2.3 Analytical validation

#### 2.3.1 Linearity and parallelism

Extracts were diluted 1:2 with purified water for use in a commercial total T3, competitive ELISA kit (MBS700042; MyBioSource Inc., San Diego, CA, USA) by following the provided kit procedure. This kit was manufactured in an ISO 9001:2015 certified laboratory, and the antibody used for this ELISA is reported to have no significant cross‐reactivity with any analogs of T3. The dilution factor for samples predicted to yield a T3 concentration beyond the 0.5–8 ng/mL detection range should be adjusted according to predicted hormone levels. Each sample was run in duplicate and randomized based on the manufacturer's recommendations and those described in ISO/IEC 17025 to ensure precision and reproducibility. The intra‐assay variability for this kit is reported to have a coefficient of variation (CV%) <15% by the manufacturer. Standards provided in the kit were entered into MyAssays Ltd. online data analysis tool (http://www.myassays.com/four‐parameter‐logistic‐curve.assay) to fit a four‐parameter logistic (4PL) curve, from which unknown concentrations were extrapolated. This same standard curve was then log‐transformed to assess linearity of the assay. An *R* ^2^ ≥ 0.95 was considered valid for this study.

An 8 ng/mL T3 standard was serially diluted to compare linearity between samples. Then, a methanol‐fecal extract was spiked with 8 ng/mL of T3 and serially diluted to assess any matrix effect due to the presence of methanol. These curves were also log‐transformed to calculate the linear slopes of each dilution. Differences of slopes were compared using the “Significance of the Difference Between Two Slopes Calculator” software available at DanielSoper.com. The CV% was also calculated for each of these slopes to confirm the parallelism of the assays. A CV% <15% was considered acceptable for our study.

#### 2.3.2 Percent recovery

Some methanol‐fecal extracts as well as human serum standards were spiked with a known concentration of 8 ng/mL of T3 before performing an ELISA to determine percent recovery of the assay. Percent recoveries of known spike concentrations were calculated using the following formula:

$$\begin{array}{c} \frac{\text{Concentratio}n_{\text{Sample with Spike}}-{\text{Concentration}}_{\text{Sample without Spike}}}{{\text{Concentration}}_{\text{Spike Alone}}}\\ \times 100=\%\text{Recovery} \end{array}$$

#### 2.3.3 Unit conversion

To convert the concentration of T3 extrapolated for 1 mL of the diluted fecal extract to the concentration of total T3 in 1 g of wet feces, the following formula can be used:

$$\frac{\mathrm{ng}\;\text{of}\;T3}{1\;\mathrm{mL}\;\text{of Diluted Extract}}\times \frac{\text{Total}\;\mathrm{mL}\;\text{of MtOH}+\text{Total}\;\mathrm{mL}\;\text{of}\;H_{2}O}{g\;\text{of}\;\mathrm{Wet}\;\text{Feces}}$$

For example:

$$\begin{array}{c} \frac{1.46\;\mathrm{ng}\;\text{of}\;T3}{1\;\mathrm{mL}\;\text{of Diluted Extract}}\times \frac{1.5\;\mathrm{mL}\;\text{of MtOH}+1.5\;\mathrm{mL}\;\text{of}\;H_{2}O}{0.01\;g\;\text{of}\;\mathrm{Wet}\;\text{Feces}}\\ =\frac{1.46\;\mathrm{ng}}{1\;\mathrm{mL}}\times \frac{3\;\mathrm{mL}}{0.01\;g}=438\frac{\mathrm{ng}}{g} \end{array}$$

### 2.4 Biological validation

#### 2.4.1 Treatment with thyroid hormones

Fifteen total mature female C57Bl/6J mice were maintained in conditions as previously described. Ten of these mice were randomly selected to receive T3 (Catalog ICN19458580; Fisher Scientific) and T4 (Catalog ICN15214501; Fisher Scientific) supplementation to model hyperthyroidism. Thyroid hormone (TH) treatment consisted of adding 2 μg/mL of T4 and 0.5 μg/mL of T3 to water containing 0.2% w/v grape Kool‐Aid sweetened with 0.4% w/v Splenda_®_ (sucralose) which was stabilized with 0.1% bovine serum albumin (BSA; Catalog BP1600‐100; Fisher Scientific) and 4 mM NaOH (Catalog S318‐500; Fisher Scientific) (Capelli et al., 2021; Niedowicz et al., 2021). The five remaining mice received sweetened Kool‐Aid including the same concentrations of BSA and NaOH without added T3 or T4 to serve as controls. After 6 weeks, five fecal pellets were collected from each animal then stored at −80°C until methanol extractions were prepared. Mice were anesthetized with isoflurane by nose cone method for several seconds, then approximately 1 mL of whole blood was collected in a heparinized (Catalog AAA16198MD; Fisher Scientific) syringe by cardiac puncture. About 500 μL of plasma was separated from whole blood and stored at −80°C. While anesthetized, mice were euthanized by cervical dislocation.

#### 2.4.2 Treatment with methimazole

Twenty‐four total 3‐week‐old female and twenty‐four total 3‐week old male C57Bl/6J mice were purchased from The Jackson Laboratory (Bar Harbor, ME, USA; RRID:IMSR_JAX:000664) and maintained in the same conditions as described previously while separated by sex and treatment group. Large experimental groups were utilized to improve statistical power while considering budget limitations. After a two‐week acclimation period, 18 random mice of each sex began treatment to model hypothyroidism. Methimazole (Catalog 11–101‐1115; Fisher Scientific) at 0.1% w/v was added to water containing 0.2% w/v grape Kool‐Aid and 0.4% w/v of Splenda_®_ for a 10‐week period (Hoefig et al., 2015; Zhou et al., 2015). The six remaining control mice of each sex received water with Splenda_®_ and Kool‐Aid alone. After 10 weeks, the previously described procedures were followed to collect feces and plasma as well as for euthanasia. Five pellets of feces were collected from each animal, and about 1 mL of whole blood was collected, resulting in approximately 500 μL of plasma.

MMI inhibits the enzyme thyroid peroxidase, which prevents biosynthesis of T3 and T4 (Davidson et al., 1978), and effectively induces hypothyroidism in rat and mouse models at a concentration of 0.1% w/v (Hoefig et al., 2015; Zhou et al., 2015). Exact doses of pharmaceuticals are often administered to rodent models by intragastric gavage. This method requires animals to be restrained while the researcher threads a flexible tube down the esophagus and into the stomach. After treatment with gavage, rats and mice can exhibit symptoms of stress, such as increased heart rate and corticosterone levels (Balcombe et al., 2004; Walker et al., 2012). The goal of this study was to employ noninvasive treatments, which is likely to minimize stress experienced by animal models. Therefore, we administered MMI to our experimental animals through drinking water. Though exact dosages ingested by mice could not be estimated for this study, a previous study determined that treatment with 0.1% w/v of MMI through water was just as effective as daily intragastric gavage with 8 mg of MMI per 100 g of body weight (Zhou et al., 2015).

### 2.5 Correlation measures

Thyroid hormone concentrations of plasma samples collected from mice were assessed using T3 and T4 ELISA assays (MyBiosource, Inc) in addition to a Rat Thyroid Magnetic Bead Panel (Millipore Sigma). A Pearson correlation coefficient was calculated to determine the direction and magnitude of correlation between measurements of T3 concentration from fecal and plasma samples collected from the same animals. For this study, a correlation coefficient (*r*) between 0.4 and 0.6 was considered a medium correlation, between 0.6 and 0.8 was considered a high correlation, and >0.8 was considered a very high correlation.

### 2.6 Statistical analysis

All results are expressed as means of duplicate experiments. Spurious outliers were identified by performing Grubbs' tests using “Outlier calculator” software available at GraphPad.com. All other analyses were performed using R Statistical Software (v4.3.1; R Core Team 2023). Data were assessed for normality using Shapiro–Wilk test with a *p* < 0.01 considered significant. Variances between data sets were assessed using *F*‐test, and differences between groups were assessed by either independent Student's *t*‐tests or Welch's *t*‐tests, with a *p* < 0.05 considered significant. Cohen's *d* was used to determine effect size of pilot project studies, with *d* > 1.0 in all experiments, and a two‐sample *t*‐test power analysis was then performed. The statistical power (1‐ꞵ) for each analysis was reported along with *p*‐value.

## 3 RESULTS

### 3.1 Analytical validation

#### 3.1.1 Percent recovery

**FIGURE 2:**
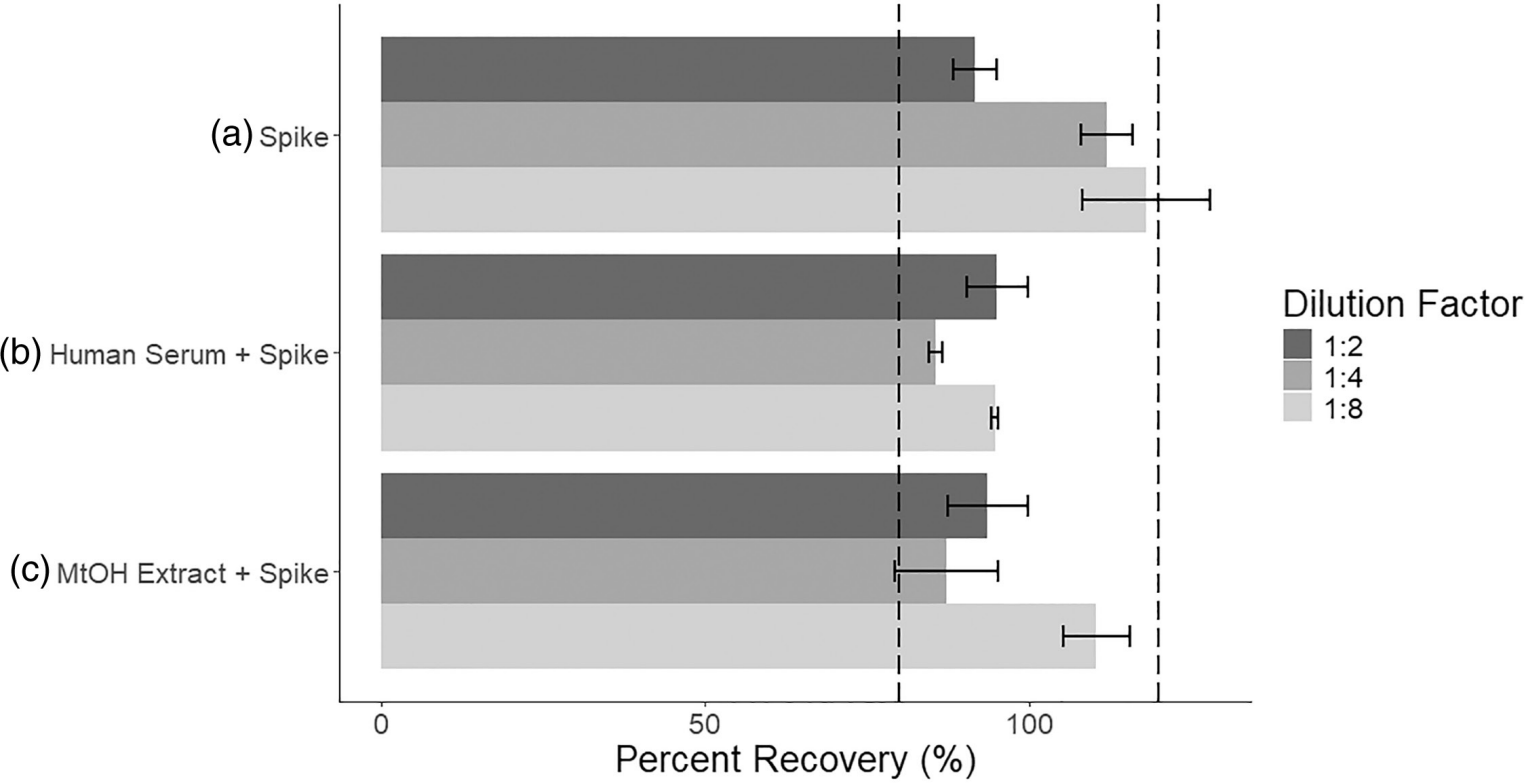
Percent recovery of T3 Standard, Spiked Human Serum, and Spiked Fecal‐Methanol Extract. (a) An 8 ng/mL T3 standard (referred to as “Spike”) was diluted 1:2, 1:4, and 1:8 with purified water. The observed T3 concentration after performing the ELISA was then divided by the expected T3 concentration to find the percent recovery of the assay. (b) Next, a human serum standard was spiked with an 8 ng/mL T3 standard (referred to as “Human Serum + Spike”). This solution was similarly diluted with purified water before use in a T3 ELISA. The T3 concentration of the human serum alone was subtracted from the observed T3 concentration of the spiked sample, then divided by the expected concentration of the spike alone to determine the percent recovery. (c) Lastly, a fecal extract was prepared with methanol and spiked with an 8 ng/mL T3 standard (referred to as “MtOH Extract + Spike”). This solution was diluted with purified water before use in a T3 ELISA. The T3 concentration of the fecal extract without a spike was subtracted from the observed T3 concentration of the spiked extract, then divided by the expected concentration of the spike alone. Bars represent mean ± SD. The region between dashed lines indicates an acceptable recovery range of 80%–120%.

The percent recovery of a known 8 ng/mL spike of T3 was within an acceptable range of 80%–120% for dilutions up to 1:8 (Table 1; Figure 2). The percent recoveries of either human serum standards or mouse methanol‐fecal extracts spiked with 8 ng/mL of T3 were maintained within the same range for dilutions up to 1:8 (Table 1; Figure 2). These findings demonstrate that any matrix effect from the presence of methanol in the assay does not greatly reduce reagent efficacy. Therefore, this assay can accurately report the T3 concentrations of blood and fecal samples.

**TABLE 1:** Percent recovery of T3 standard, spiked human serum, and spiked fecal extracts.

Percent recoveries (%)			
Dilution ratio	Water +8 ng/mL T3 spike	Human serum +8 ng/mL T3 spike	Fecal extract +8 ng/mL T3 spike
1:2	91.7 ± 3.4^a^	95.1 ± 4.8^a^	93.6 ± 6.2^a^
1:4	112.0 ± 3.9^a^	85.6 ± 1.1^a^	87.3 ± 7.9^a^
1:8	118.1 ± 9.9^a^	94.7 ± 0.6^a^	110.4 ± 5.1^a^

*Note*: Both human serum and fecal extracts were spiked with 8 ng/mL T3 to determine the efficacy of the ELISA. The percent recoveries are reported as mean ± SD.

^a^ Indicates a mean percent recovery within an acceptable range of 80%–120%.

#### 3.1.2 Linearity and parallelism

**FIGURE 3:**
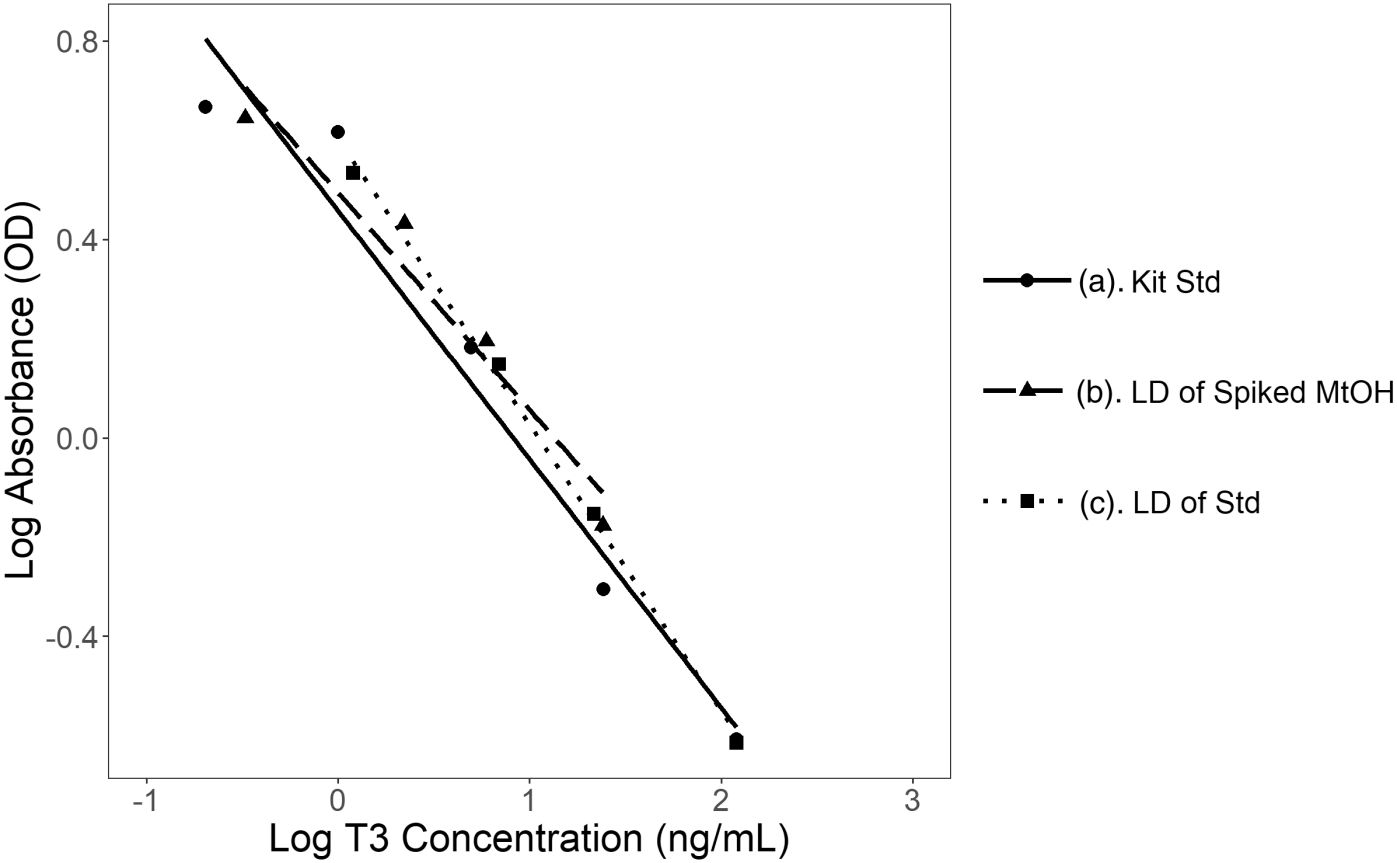
Assessing linearity and parallelism of T3 ELISA. (a) Log‐transformed standard curve created using standards provided with kit (“Kit Std,” *R* ^2^ = 0.9565). (b) Log‐transformed serial dilution of methanol‐fecal extract spiked with 8 ng/mL (“LD of Spiked MtOH,” *R* ^2^ = 0.9522). (c) Log‐transformed serial dilution of 8 ng/mL standard (“LD of Std,” *R* ^2^ = 0.9971). Data points represent the mean of duplicate wells.

The log‐transformed standard curve created using the standards provided with the T3 ELISA kit created an *R* ^2^ = 0.9565, demonstrating acceptable linearity of the assay. The log‐transformed serial dilution of the most concentrated standard, 8 ng/mL of T3, had an *R* ^2^ = 0.9971, further validating the assay's linearity. Finally, the log‐transformed serial dilution of a methanol‐fecal extract spiked with 8 ng/mL also confirms linearity of the assay when a methanol matrix is introduced, with an *R* ^2^ = 0.9522. This again supports the previous findings that the presence of methanol does not influence the validity of the assay (Figure 3). The slopes calculated for all three standard and dilution curves demonstrate parallelism of the assay, as there are no significant differences between these slopes (*p* = 0.130–0.528). The coefficient of variation (%CV) for the slopes was calculated to be 13.68%, which is within acceptable range of <15%.

### 3.2 Biological validation

#### 3.2.1 Thyroid hormone treatment

**FIGURE 4:**
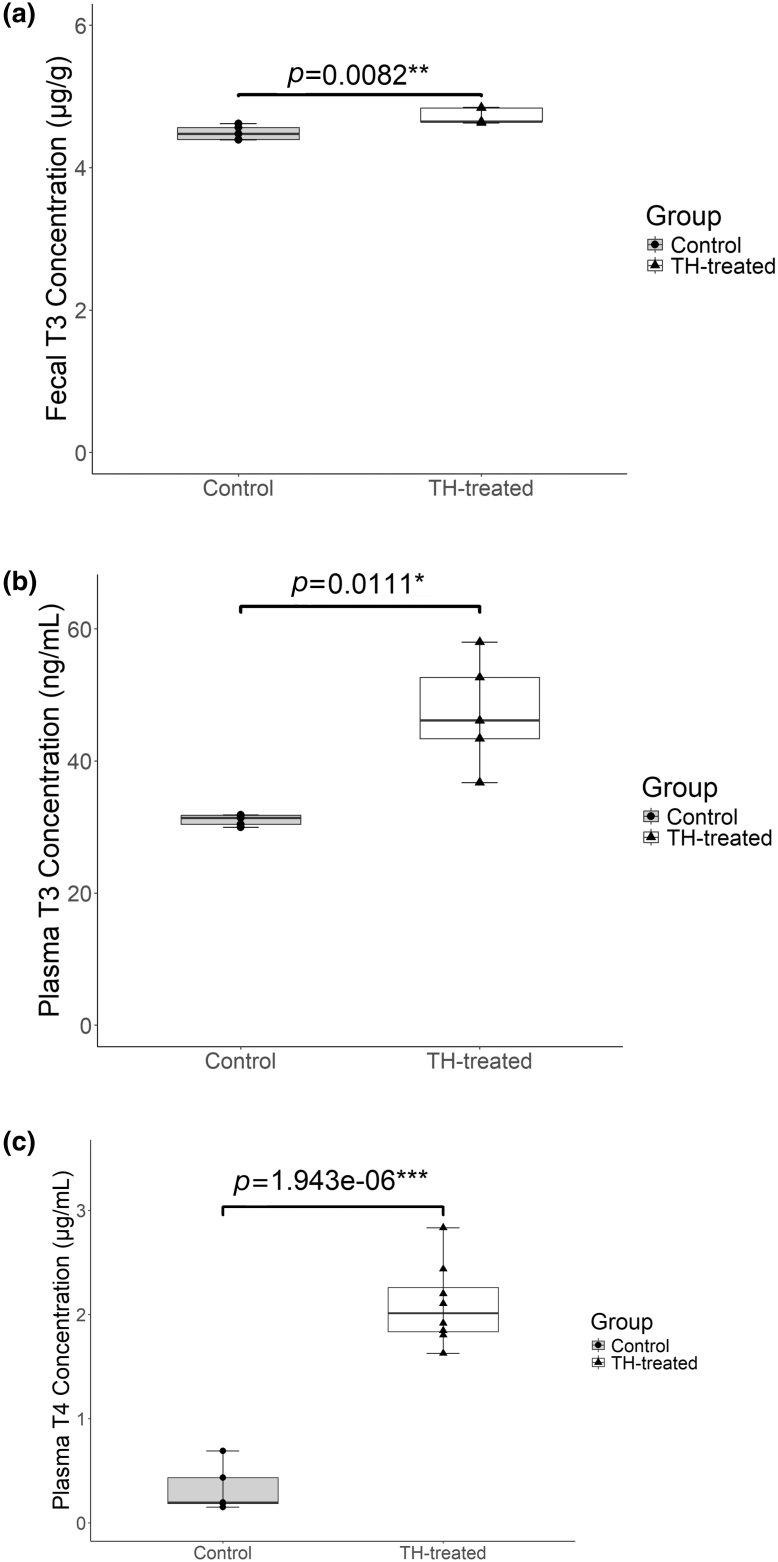
Fecal and plasma thyroid hormone concentrations in female mouse models treated with T3 and T4. (a) Fecal samples were collected from control (*n* = 5) and thyroid hormone‐treated (“TH‐treated”; *n* = 5) mice. The optimized T3 extraction protocol was used to create methanol‐fecal extracts that were then diluted 1:50 with purified water before use in a T3 ELISA kit (*p* = 0.0082, 1‐ꞵ = 0.86). (b) Plasma samples were collected from control (*n* = 5) and thyroid hormone‐treated (“TH‐treated”; *n* = 5) mice then diluted 1:50 with purified water before use in a T3 ELISA kit (*p* = 0.0111, 1‐ꞵ = 0.97). (c) Plasma samples were collected from control (*n* = 5) and thyroid hormone‐treated (“TH‐treated”; *n* = 8) mice then diluted 1:50 with purified water before use in a T4 ELISA kit (*p* = 1.943e‐06, 1‐ꞵ = 1.0). Data points represent the mean of duplicate wells. Boxes represent the interquartile range (IQR) from Q1 to Q3, horizontal lines represent the median, and whiskers represent Q1–1.5× IQR and Q3 + 1.5× IQR. (*p < 0.05, **p < 0.01, ***p < 0.001).

Methanol‐fecal extracts were prepared from female mice administered T4 and T3 orally for 6 weeks. Extracts from control and experimental mice were diluted 1:50 with purified water before use in the T3 ELISA, as these samples were predicted to exceed the detection range of the kit. Mice treated with thyroid hormones excreted significantly more T3 per gram of feces relative to control mice (Figure 4a).

To verify that T4 and T3 treatment increased thyroid hormone levels beyond those of control mice, plasma samples were diluted 1:50 with purified water and utilized in a T3 ELISA and a T4 ELISA. Treated mice had significantly higher levels of circulating total T3 and T4 than those of control mice (Figure 4b,c).

#### 3.2.2 Methimazole treatment

Differences between control and thyroid hormone‐treated mice were detectable from fecal T3 measurements alone. Next, a similar experiment was designed to reduce thyroid hormone levels below that of control mice through oral administration of methimazole (MMI). This experiment was expanded to include both female and male mice to ensure that the technique of fecal T3 measurement can be implemented for either sex.

**FIGURE 5:**
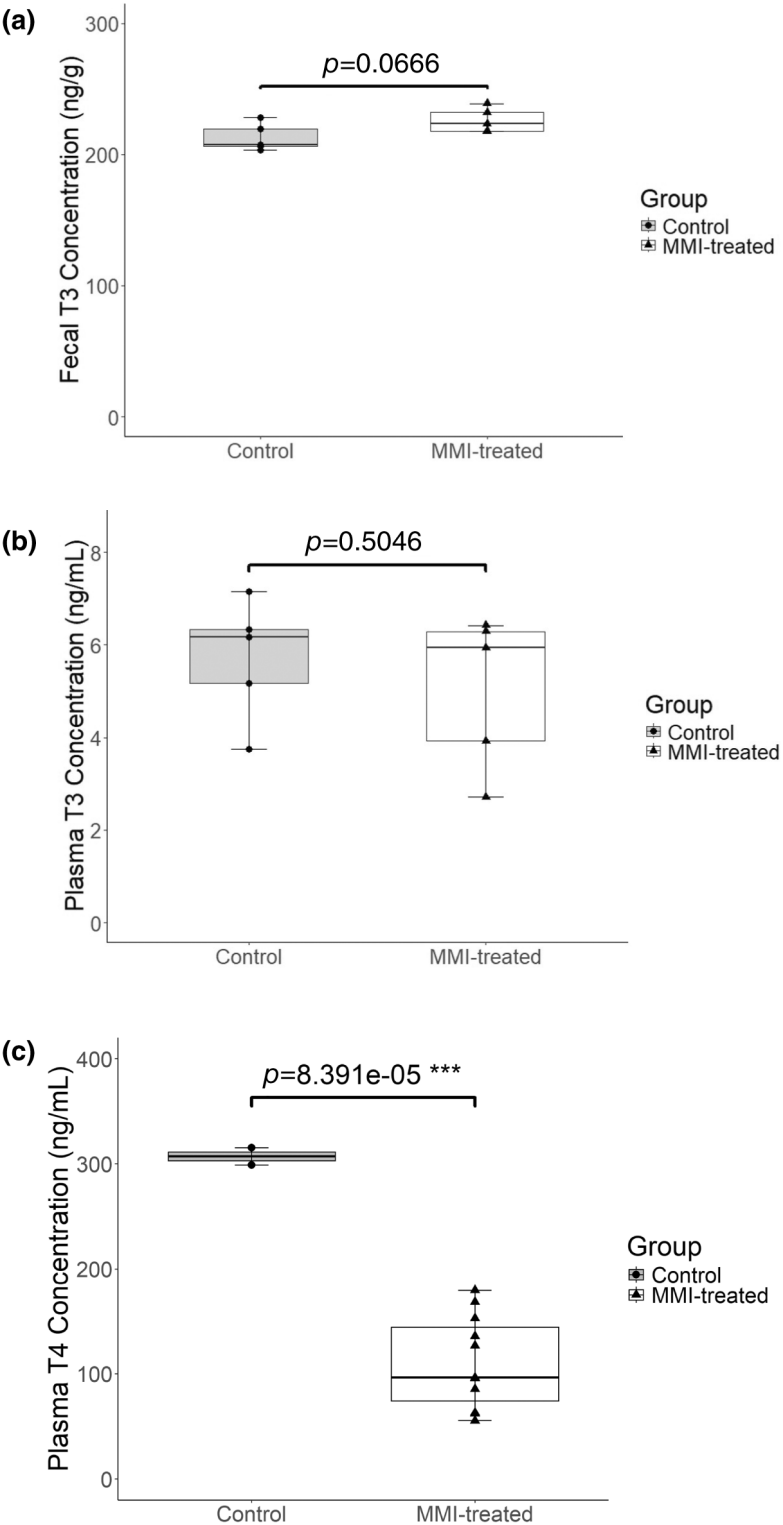
Fecal and plasma thyroid hormone concentrations of methimazole‐treated female mouse models. (a) Fecal samples were collected from control (*n* = 5) and methimazole‐treated (“MMI‐treated”; *n* = 5) mice. The optimized T3 extraction protocol was used to create methanol‐fecal extracts that were then diluted 1:2 with purified water before use in a T3 ELISA kit (*p* = 0.0666, 1‐ꞵ = 0.46). (b) Plasma samples were collected from control (*n* = 5) and methimazole‐treated (“MMI‐treated”; *n* = 5) mice then used in a T3 ELISA kit (*p* = 0.5046, 1‐ꞵ = 0.09). (c) Plasma from control (*n* = 2) and methimazole‐treated (“MMI‐treated”; *n* = 11) mice was diluted 1:6 before use in a thyroid magnetic bead panel to assess circulating total T4 (*p* = 8.391e‐05, 1‐ꞵ = 1.0). Data points represent the means of duplicate wells. Boxes represent the interquartile range (IQR) from Q1 to Q3, horizontal lines represent the median, and whiskers represent Q1–1.5× IQR and Q3 + 1.5× IQR. (***p < 0.001).

**FIGURE 6:**
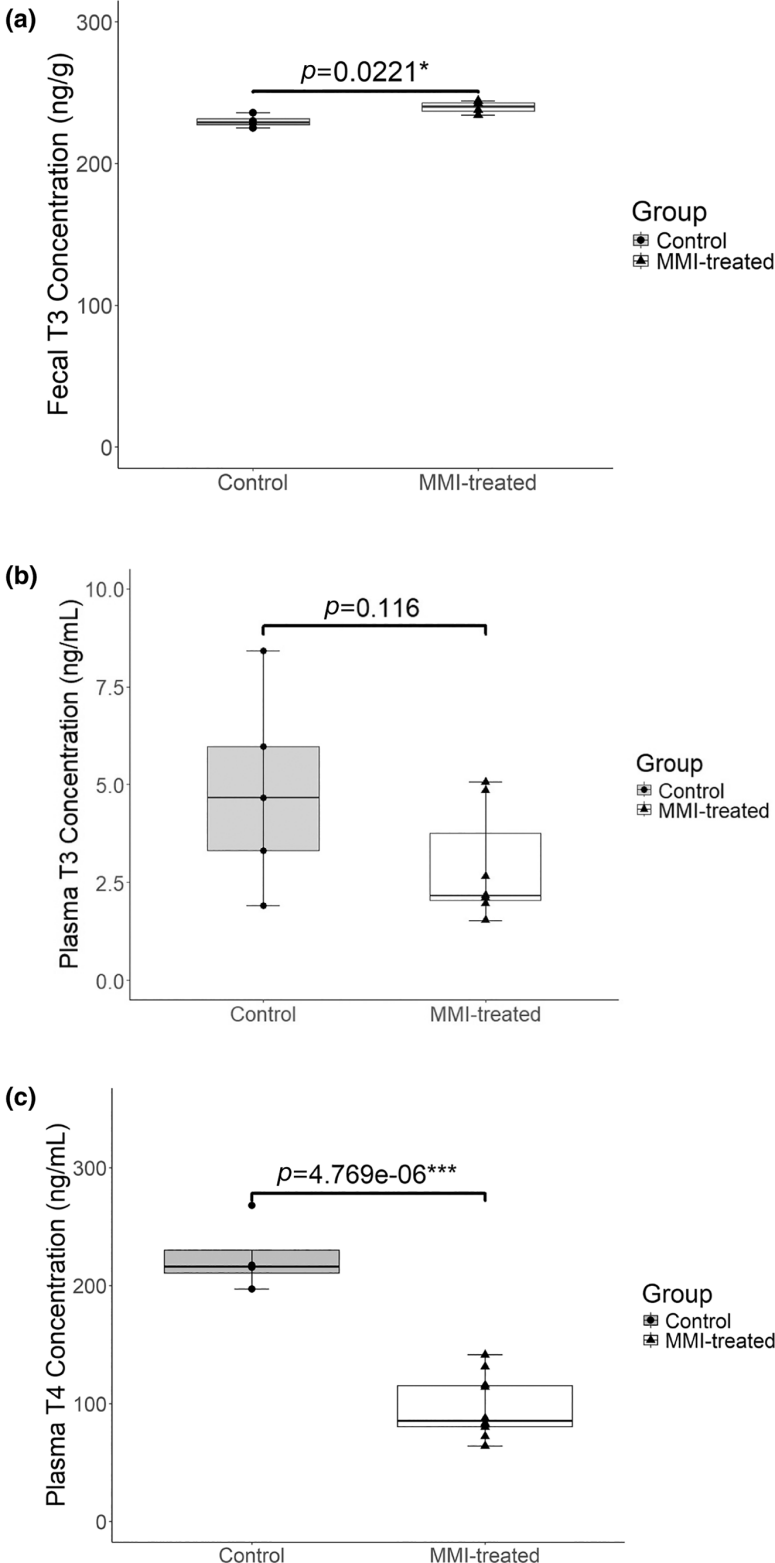
Fecal and plasma thyroid hormone concentrations of methimazole‐treated male mouse models. (a) Fecal samples collected from control (*n* = 4) and methimazole‐treated (“MMI‐treated”; *n* = 4) mice. The optimized T3 extraction protocol was used to create methanol‐fecal extracts that were then diluted 1:2 with purified water before use in a T3 ELISA kit (*p* = 0.0221, 1‐ꞵ = 0.72). (b) Plasma samples were collected from control (*n* = 5) and methimazole treated (“MMI‐treated”; *n* = 7) mice and used in a T3 ELISA kit, then diluted 1:6 and used in a thyroid magnetic bead panel (*p* = 0.116, 1‐ꞵ = 0.34). (c) Plasma samples were collected from control (*n* = 4) and methimazole treated (“MMI‐treated”; *n* = 10) mice and used in a T4 ELISA kit, then diluted 1:6 and used in a thyroid magnetic bead panel (*p* = 4.769e‐06, 1‐ꞵ = 1.0). Data points represent the mean of duplicate wells. Boxes represent the interquartile range (IQR) from Q1 to Q3, horizontal lines represent the median, and whiskers represent Q1–1.5× IQR and Q3 + 1.5× IQR. (*p < 0.05, ***p < 0.001).

Methanol extractions were prepared using the fecal samples collected from control and experimental mice. These extracts were diluted 1:2 with purified water before use in the T3 ELISA. MMI‐treated female mice excreted slightly more T3 per gram of feces relative to the control mice, though this difference was not found to be significant (*p* = 0.0666, Figure 5a). These data exhibited a high effect size, yet low statistical power, suggesting that the sample size was not sufficiently large to reflect the biological variability of the female animals. When the same treatment was assessed in male mice, it was found that the MMI‐treatment significantly increased the excretion of T3 per gram of feces relative to control mice (Figure 6a).

Plasma collected from both female and male mice were used in a T3 ELISA to determine circulating total T3 levels. These findings suggest there was no difference in circulating T3 between control and experimental mice of either sex (Figures 5b and 6b), though each of these analyses had low statistical power. This finding is not entirely unexpected, as the HPT axis and peripheral tissues may adjust expression of certain genes, such as *TSH* and *Dio2*, to maintain a “normal” circulating T3 concentration (Abdalla & Bianco, 2014).

In line with the challenges that prompted the development of this protocol, there was insufficient plasma to assess total circulating T4 levels by ELISA, so instead a more sensitive but more costly Rat Thyroid Magnetic Bead Panel was used. To test for inter‐assay variation, samples previously analyzed in the MyBiosource ELISAs were tested using the thyroid panel kit and were found to be within ±1% of the ELISA values. Experimental female and male mice exhibited significantly reduced levels of circulating total T4 relative to control mice, confirming that the reduction of thyroid hormone levels in experimental mice compared to controls was statistically significant (Figures 5c and 6c).

### 3.3 Fecal and plasma T3 correlation

**FIGURE 7:**
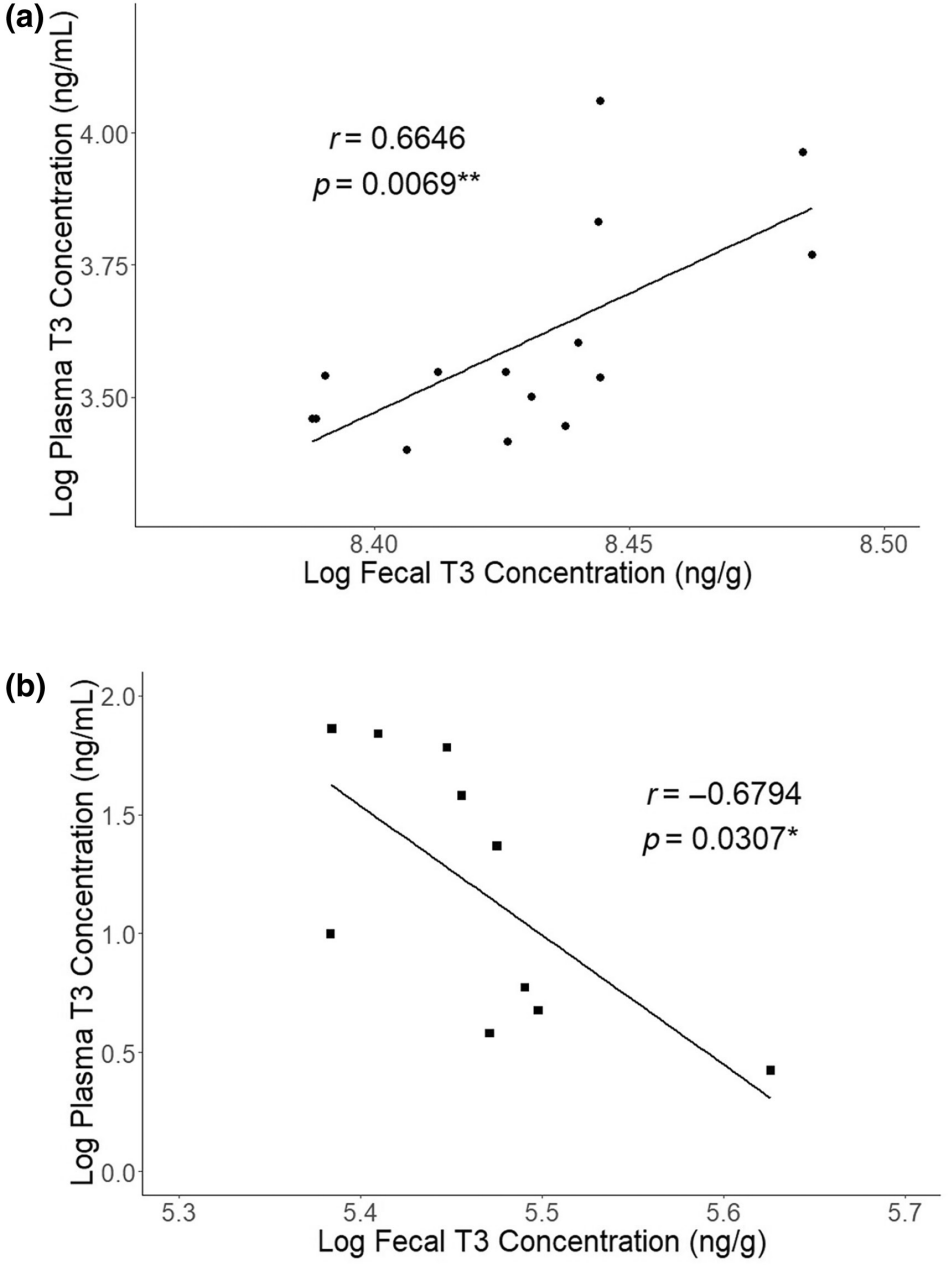
Correlation of fecal and plasma T3 measurements. (a) When mice are treated with thyroid hormones (*n* = 15), there is a strong, positive correlation between excreted T3 measured in feces and circulating T3 measured from plasma (*r* = 0.6646, *p* = 0.0069). (b) When mice are treated with methimazole (*n* = 10), there is a strong, negative correlation between excreted T3 measured in feces and circulating T3 measured from plasma (*r* = −0.6794, *p* = 0.0307). Data points on the bivariate plots represent means of duplicate wells, and trendline represents linear model fit to data points. (*p < 0.05, **p < 0.01).

Both fecal and plasma T3 levels were elevated in thyroid hormone‐treated mice. To characterize the specific relationship between these measurements, fecal and plasma T3 measurements from the same animals were log‐transformed and a Pearson correlation coefficient was calculated. A strong, positive correlation between fecal and plasma total T3 was discovered (Figure 7a). During a thyroid hormone‐treated state, fecal, and plasma T3 measurements are directly related.

This direct relationship between fecal and plasma T3 levels was not observed in methimazole‐treated mice. Instead, the fecal T3 concentrations slightly increased, similarly to mice treated with thyroid hormones (Figures 5a and 6a). When the Pearson correlation coefficient for fecal and plasma T3 measurements of MMI‐treated mice was calculated, a strong, negative correlation between fecal and plasma T3 was observed (Figure 7b). Thus, during a methimazole‐treated state, fecal and plasma T3 measurement are inversely related, rather than directly.

## 4 DISCUSSION

While laboratory mice are well‐established models for human physiology, a major limitation for hormone analysis is the ability to easily acquire sufficient blood to measure multiple hormones within a relatively short time frame. An attractive alternative is the use of non‐invasive sampling protocols such as fecal sampling for hormone analysis. There is a paucity of published research utilizing or validating a technique for measuring fecal T3 or T4 in laboratory mice.

When considering a nontraditional approach for thyroid hormone analysis, a major challenge presents itself in the interpretation of the physiological relevance of the data. To the best of our knowledge, no comparison data for fecal T3 or T4 levels in mice exists. A full understanding of physiological changes in fecal thyroid hormones will take time and the accumulation of a larger data set. Thus, more work is needed to correlate changes in fecal thyroid hormone levels with the well‐established picture of changes in plasma levels.

While measuring T3 and T4 levels in feces is readily accomplished due to their lipophilic nature, it is our understanding that measurement of thyroid stimulating hormone (TSH), a peptide hormone, in feces has not been reported. Because TSH is the hormone that stimulates synthesis and secretion of T3 and T4, a full picture of thyroid function cannot be achieved through analyzing only fecal samples. As in humans, measurement of TSH levels in mice is important for understanding T3 and T4 levels. In other words, TSH is the principal regulator of the thyrostat, so for the time being, at least some blood sampling is still necessary. Additionally, if researchers would like to use this protocol to measure T3 in fecal samples using a different ELISA, the new ELISA would need to be validated. Furthermore, for repeated sampling over time in individual mice, tagging or marking the animals would be required.

On the other hand, there are major benefits to utilizing fecal sampling. Compared with blood volume, the amount of feces a mouse can produce per day is astronomical. Thus, there is no real limit on the amount of sample that can be collected, and it could be collected multiple times per day. Another desirable aspect of this technique is the ease with which samples can be collected. This is especially advantageous for research involving undergraduates or researchers new to the mouse model. Finally, there are benefits from the nature of the sample itself: multiple lipophilic hormones can be measured in a single sample because of the large quantity available at a given time. The samples are quite stable and do not require immediate processing upon collection (unlike blood) and the extraction process is relatively quick.

Our validated assay for noninvasive monitoring of fecal T3 levels in either sex of laboratory mice prioritizes use of accessible and affordable reagents. Using this assay, we were able to identify differences in fecal T3 levels between control mice and mice treated with either thyroid hormones or methimazole. Plasma concentrations of total T3 and T4 confirmed these observed differences, which also confirms that a directional correlation exists between fecal and plasma T3 levels. Thus, fecal samples can effectively detect fluctuations in the thyroid system.

## AUTHOR CONTRIBUTIONS

Cinnamon L. VanPutte and Lucia M. Thompson conceived and designed research, performed experiments, analyzed data, interpreted results of experiments, prepared figures, drafted manuscript, edited and revised manuscript. Brailey M. Coulter performed experiments and analyzed data. Cinnamon L. VanPutte approved final version of manuscript.

## FUNDING INFORMATION

SIUE URCA Associate Scholarship (to LT). SIUE Seed Grants for Transitional and Exploratory Projects (STEP) grant and SIU SDM Advanced Initiative Award (AIA23‐02; to CVP).

## CONFLICT OF INTEREST STATEMENT

The authors declare no conflicts of interest.

## ETHICS STATEMENT

Animal care procedures and experiments designed for this study were approved by the Institutional Animal Care and Use Committee of Southern Illinois University Edwardsville (IACUC protocols #1167/2570). The experiments described were conducted in compliance with the US National Research Council’s “Guide for the Care and Use of Laboratory Animals,” the US Public Health Service’s “Policy on Humane Care and Use of Laboratory Animals” and “Guide for the Care and Use of Laboratory Animals.”

## Data Availability

The data that support the findings of this study are available from the corresponding author upon reasonable request.
